# A Simple Non-invasive I-123-IMP Autoradiography Method Developed by Modifying the Simple Non-invasive I-123-IMP Microsphere Method

**DOI:** 10.22038/aojnmb.2017.9555

**Published:** 2018

**Authors:** Asato Ofuji, Rieko Nagaoka, Kosuke Yamashita, Akihiro Takaki, Shigeki Ito

**Affiliations:** 1 Graduate School of Health Sciences, Kumamoto University, Kumamoto, Japan; 2 National Hospital Organization Kyushu Medical Center, Fukoka, Japan; 3 Fujifilm RI Pharma Co., Ltd., Tokyo, Japan; 4 Faculty of Life Sciences, Kumamoto University, Kumamoto, Japan

**Keywords:** Auto-radiography (ARG) method, ^123^I-IMP, Non-invasive method, rCBF, SPECT

## Abstract

**Objective(s):**

We developed a simple non-invasive I-123-N-isopropyl-p-iodoamphetamine (^123^I-IMP) quantification method by analyzing chest RI-angiography and single photon emission computed tomography (SPECT) images based on the microsphere model (SIMS method). Theoretically, the SIMS method could be changed to the simple non-invasive ARG (SIARG) method by modifying the integrated washout ratio (WR) to one-point WR. If the regional cerebral blood flow (rCBF) values derived from the SIARG and ARG methods correlate well, the facilities employing the ARG method can easily switch to the SIARG method. The purpose of this study was to develop the SIARG method by modifying the SIMS method, and to confirm the feasibility of this method.

**Methods:**

The correlation between the input counts of the SIARG method and the blood counts was determined by linear regression analysis. The rCBF values determined by the SIARG method were compared with the values obtained with the ARG and SIMS methods.

**Results:**

There was a good linear correlation between the SIARG counts and the arterial blood counts obtained by the ARG method (r=0.85, P<0.001, n=29). The rCBF values obtained by the ARG and SIARG methods (n=29, 696 ROIs) correlated well (y=1.01x – 3.6, r=0.85, P<0.001). Similarly, the rCBF values obtained by the SIARG and SIMS methods (n=29, 696 ROIs) correlated well (y=0.98x – 15.2, r=0.90, P<0.001). rCBF values obtained by the SIARG method were almost the same as the values obtained by the ARG method, and values of the SIMS method were 15 ml/100g/min higher that of those obtained by the SIARG method.

**Conclusion:**

The rCBF values obtained by the ARG, SIARG, and SIMS methods correlated very well. Therefore, the SIARG method is potentially useful for examinations in routine clinical practice.

## Introduction

We developed a simple non-invasive I-123-N-isopropyl-p-iodoamphetamine (^123^I-IMP) quantification method by analyzing chest RI-angiography and single photon emission computed tomography (SPECT) images based on the microsphere model with the two-dimensional ordered subset expectation maximization (2D-OSEM), Chang’s attenuation correction, and the scatter correction (the triple energy windows method: TEW)) (SIMS method) (1, 2). The regional cerebral blood flow (rCBF) values of the SIMS were approximately equal to the continuous arterial blood sampling measurements and measurements of the octanol extraction fraction of the blood obtained by the microsphere model method (MS method) (1, 3-5).

Due to its convenience, the ^123^I-IMP autoradiography (ARG) method based on a 2-compartment model, instead of the MS method, is currently used widely in clinical practice in Japan (6-13). The rCBF values obtained by the MS method with filtered back projection algorithm (FBP) are approximately 20% higher than those obtained by the ARG method with FBP (8). When facilities switch from the ARG method to the SIMS method, discrepancies of the rCBF values between the ARG and SIMS methods may lead to problems with the diagnosis due to different quantification theory for the rCBF values (3, 6). Therefore, it is necessary to clarify the correlation between the rCBF values obtained by the ARG and SIMS methods.

The input function of the ARG method is estimated by single-point arterial blood sampling and the standard input function (6, 7). The input function of the SIMS method was automatically determined by using the area under the curve (AUC) of the pulmonary artery (PA) and the integrated lung washout ratio (WR) which were obtained by analyzing results from chest RI-angiography (1, 2). Theoretically, since the blood counts of the ARG method should be directly proportional to the product of the simple non-invasive ARG (SIARG) method using the AUC and one-point WR, the SIMS method could be changed to the SIARG method by modifying the integrated WR to a one-point WR. If a good correlation between the SIARG and ARG methods in rCBF values is found, the facilities employing the ARG method can easily switch to the SIARG method. Additionally, the rCBF values can be converted from the SIARG method to the SIMS method using a fully automatic program.

The purpose of this study was to develop the SIARG method by modifying the SIMS method and to confirm the feasibility of this method.

## Methods

### Theory

rCBF values of the ARG method are calculated as follows:


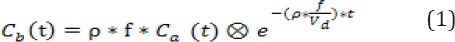


where *C*(_*b*_)*(t)* (kBq/ml) is the radioactivity concentration in the brain at time t, *C*(_*a*_)*(t)* is the arterial input function at time t, ⊗ is the convolution integral, ρ (1.04 g/ml) is the density of the brain tissue, f (ml/100g/min) is rCBF, and V_d_ is the regional distribution volume (6).

On the other hand, rCBF values of the SIMS method are calculated as follows:


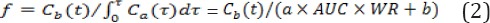


where *C*(_*a*_) (*τ*) (kBq/ml) is the arterial input function. The constants *a* and *b* can be estimated by linear regression analysis (1).

**Figure 1:**
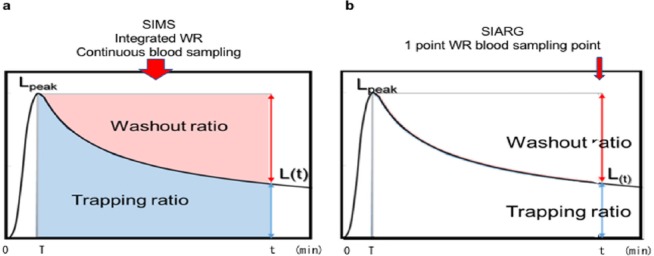
The count-time-activity curve (TAC) of the lung


The AUC of the count-time activity was obtained by integrating the gamma functions. The lung washout ratio (WR) was determined by analyzing the time activity curve (TAC) (Figure 1).

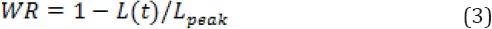


where *L_peak_* is the peak radioactivity of the lung and *Ly_(t)_* is radioactivity of the lung at time t corresponding to the arterial blood sampling time.

We hypothesized that the arterial input function reflects the administration and one-point clearance of ^123^I-IMP from the lung. When the WR is obtained by one-point time corresponding to the arterial blood sampling time, the (*a*×*AUC*×*WR*+*b)* is equal to the *C*(_*a*_) *(t)* in the Eq. (1).

Therefore, rCBF values can be calculated noninvasively based on the ARG method with ^123^I-IMP.

### Subjects

None of the patients had severe cardiac disease, pulmonary disease and smokers. The studies were approved by the institutional ethics boards of the National Hospital Organization Kyushu Medical Center and Kumamoto University Hospital, and written informed consent was obtained from all patients before the study began. All image data were handled anonymously, in accordance with the Declaration of Helsinki and the regulation of each institutional ethics board.

The images of 29 consecutive patients (21 men, 8 women; age range=53-82 years; mean age=67.5 years) who underwent both ^123^I-IMP chest RI angiography and SPECT examinations at the same time (^123^I-IMP examinations) were used for the SIARG method development. The clinical diagnoses of the patient population are listed in Table 1. The first 17 patients date were used for the theory and procedure building of the SARG method. Seven out of the 17patiens were acetazolamide (ACZ) charge test and 10 patients were resting state. For validation of the accuracy of the SIARG method, the images of All patients were used, and 13 patients were ACZ challenge tests.

**Table 1:** Clinical diagnoses of subjects

Disease	Number of patients
ICA^1)^ stenosis	17
MCA^2)^ stenosis	4
VA^3)^ stenosis	1
Moyamoya disease	6
Epilepsy	1
Total	29

1) Internal carotid artery: ICA

2) Middle cerebral artery: MCA

3) Vertebral artery: VA

### ^123^I-IMP imaging protocol

^123^I-IMP imaging was performed by using a dual-head SPECT scanner (E.cam, SIEMENS, Germany). ^123^I-IMP dynamic planar images of the chest in the anterior view were obtained for 2 min (1 s/frame, 128×128 matrix, 4 mm/pixel) using a detector equipped with low-energy high-resolution (LEHR) collimators after a bolus injection of 222 MBq of ^123^I-IMP. Three energy windows were selected for the projection data; the main window had a width of 20% (32 keV) and was centered at 157 keV, while the two additional windows above and below the main window had a width of 7% (11 keV) for triple-energy window (TEW) scatter correction.

SPECT was performed at the 25-min mid-scan time using a scanner equipped with LEHR collimators. Projection data were acquired every 20 s by continuously rotating the detector by 360° (60 steps/360°/20 s, 128×128 matrix). SPECT images were obtained using the ordered subsets expectation maximization (OSEM) method (subsets 4, iterations 25). An attenuation correction was performed using Chang’s method, with the outline extracted from the head surface by the 15% threshold of the SPECT images. An attenuation coefficient of 0.15/cm and a Butterworth filter (cut-off 0.5 cycles/cm, order 8) was used for the image reconstruction.

### CBF analysis (SIARG vs. ARG)

The correlation between the input counts of the SIARG method and the blood counts was determined by linear regression analysis. The input counts for the SIARG method was calculated using a regression equation.

All SPECT images, after being converted by the Statistical Parametric Mapping 8 (SPM8; Wellcome Department of Cognitive Neurology, Institute of Neurology, London, UK), were analyzed using a three-dimensional (3D) stereotaxic ROI template (SRT) on anatomically standardized CBF SPECT images for objective estimation of the rCBF (14). The 3DSRT is composed of 528 ROIs in 24 segments of the anterior, precentral, central, parietal, angular, temporal, occipital, pericallosal, lenticular nucleus, thalamus, hippocampus, and cerebellar regions of the brain on each side. The rCBF values were defined as the mean values for each segment.

The rCBF values determined by the SIARG method were compared with the rCBF of the ARG method. The rCBF values by the ARG and SIARG methods were obtained using Eq.(1) and Eq.(2), respectively. The V_d_ values for the ARG and SIARG methods were fixed at 35 ml/ml for all image reconstructions (6-8).

The input counts of the SIARG method were automatically determined by modifying the fully automatic SIMS program (2). In the SIMS method, the TAC of the PA was obtained by placing a circular ROI with the maximum counts on the PA among all the dynamic images. The AUC of the first peak was obtained by fitting with the gamma function. The TAC of the lung for the WR was obtained by setting the lung ROI in such a location that it was not piled up on the subclavian veins, cephalic veins, or the superior vena cava (SVC). The mean value in both TAC of the lungs was defined as the WR. The TAC at 10 min after injection was estimated by fitting an exponential function (1, 2). The WR at the 10 min for the SIARG method was calculated by using the Eq. (3). The input counts of the SIARG method was obtained by multiplying the AUC to WR.

For the ARG method, one-point arterial blood sampling was performed 10 min after ^123^I-IMP infusion. The radioactivity of the blood sample was measured by a well-type NaI scintillation counter.

### Stastical analysis

The correlation between the ARG and SIARG CBF values were graphically plotted and the regression parameters (regression slope, regression y-intercept, and correlation coefficient) were statistically compared. Statistical significance for all analyses was set at a probability value of less than 0.05. The Bland-Altman method was used to confirm the mean difference between the ARG, SIARG, and SIMS values. Analyses were performed using MedCalc software (version 12.4, MedCalc Software bvba, Ostend, Belgium).

## Results

Figure 2a shows the correlation between the SIARG input counts and the arterial blood counts for the ARG method. A good linear correlation was obtained between the SIARG counts and the arterial blood counts of the ARG method (r=0.95, P<0.001, n=17). The regression equation for the input function of the SIARG method was determined as follows:

**Figure 2a:**
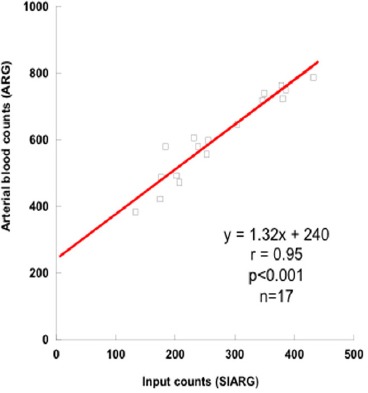
Correlation between the simple non-invasive ARG (SIARG) input counts and the arterial blood counts of the ARG method. The regression equation obtained for determining the Ca(t) was: y = 1.32*x* + 240 (r=0.95, P<0.01)


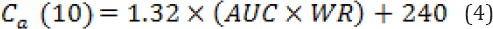


The input function for the SIARG method was obtained using Eq. (4).

A good linear correlation was also obtained between the SIARG counts and the arterial blood counts of the ARG method in all subjects (r=0.91, P<0.01, n=29) (Figure 2b). The regression equation was approximately equal to the Eq. (4).

**Figure 2b:**
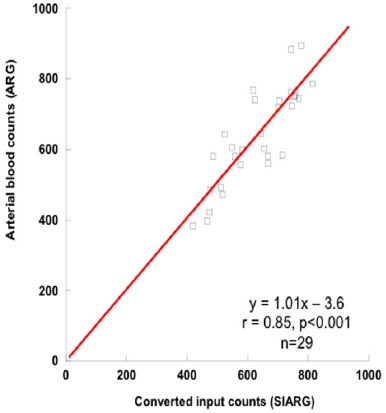
Correlation between the simple non-invasive ARG (SIARG) input counts and the arterial blood counts of the ARG method

**Figure 3:**
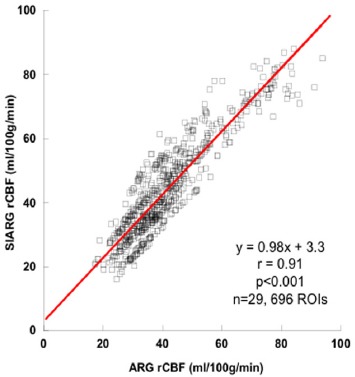
Correlation between the ARG regional cerebral blood flow (rCBF) and SIARG rCBF

**Figure 4:**
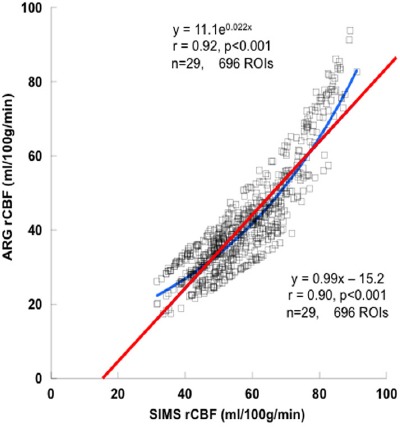
Correlation between the ARG regional cerebral blood flow (rCBF) and SIMS rCBF

Figure 3 shows that the rCBF values obtained by the ARG and SIARG methods (n=29, 696 ROIs) correlated well (y=0.98x + 3.3, r=0.91, P<0.001). Similarly, the rCBF values obtained by the SIMS and ARG methods (n=29, 696 ROIs) correlated well (y=0.99x – 15.2, r=0.90, P<0.001) as shown in Figure 4.

rCBF values of the SIARG method were approximately equal to the values of the SIMS method in 30-80 ml/100g/min.

## Discussion

The SIARG method consists of changing the integrated washout ratio (WR) of the lung in the SIMS method to the one-point WR based on the arterial blood sampling time of the ARG method.

The SIARG method was developed by modifying the SIMS method in order to compensate discrepancies of the rCBF values between the ARG and SIMS methods (1). Difference between the SIARG and ARG methods is only the arterial input counts determination process by the image analyzing or arterial blood sampling. There are two approaches to apply the SIARG method in clinical study. First of all, the input counts of the SIARG method can be easily converted by using the count conversion coefficient between the gamma camera and well-type scintillation counter in order to estimate the arterial blood counts as shown in Figure 2. Secondly, since the SIARG method is required RI-angiography counts (2-dimensional (2D) planar counts) and SPECT counts (3-dimensional (3D) counts), a 2-3D count conversion factor should be experimentally determined by using a cylindrical phantom filled in the ^123^I solution (1, 15). Alternatively, the regression equation for the constants of the Eq. (4) could be determined by analyzing the arterial blood counts of the ARG data and the AUC counts of the Chest RI-angiography of 10-20 patients. The sensitivity of the gamma camera will be experimentally determined by using a cylindrical phantom.

The theory of the ARG method based on the 2-compatment model is different from that of the MS method based on the microsphere model as shown in Eq. (1) and (2) (1, 6). The rCBF values on the 3DSRT were used to clarify the influence on these different models in this study. The arterial blood sampling counts of ARG method and the input counts of SIARG have good correlation (Figure 2). rCBF values obtained by the ARG and SIARG methods correlated very well (Figure 3). The rCBF values of the SIARG method were approximately equal to those values of the ARG method. Therefore, we anticipate that the SIARG method will easily replace the ARG method in many clinical facilities.

The rCBF values obtained by the ARG and SIMS methods correlated very well (Figure 4). However, this relationship was expressed by linear and exponential regression equations. This reason can be caused by the difference of the theory between the ARG and the MS method (1, 6). The rCBF values of the SIARG method could be converted to those values of the SIMS method by using an exponential regression equation. However, rCBF values obtained by the SIARG method were almost the same as the values obtained by the ARG method in 30-80 ml/100g/min. This means that the rCBF values of the SIARG method should be applied during clinical practice without conversion to those values of the SIMS method. These results of this study were obtained from 29 patients. Therefore, further investigations using many subjects in other facilities are required for the clinical routine study, since correlation analyses depend on the number of samples.

Tomimatsu et al. reported a new regression equation for ^123^I-IMP non-invasive cerebral blood flow measurement using a graph plot (GP) method (16). Additionally, Kaminaga et al. reported the correlation CBF values between the non-invasive microsphere (NIMS) method and ARG method (17). These methods require planar brain and chest RI-angiography and planar images (16, 17). The brain counts of the planar image for these methods are underestimated due to gamma-ray absorption by bone and brain tissues, although input counts are obtained by ^123^I input counts of these methods are equal to those of the SIARG method. Thus, rCBF values of the GP and NIMS methods were approximately 30-40% lower than those of the ARG method (16, 17). However, the rCBF values of the SIARG method were approximately equal to the ARG method (Figure 3). For this reason, the SIARG method can be superior to the GP and NIMS methods.

The best way for validation of the SIARG method would be to compare with the H_2_^15^O PET method as the golden standard (18, 19). However, it was difficult to determine the rCBF by both the SIARG and H_2_^15^O PET method simultaneously for each patient in this study. The correlations of the rCBF values obtained by the ARG and H_2_^15^O PET method have already been clarified by many studies (13, 16, 19, 20). For this reason, the CBF values of the SIARG method was compared with the ARG method as the standard, which is the limitation of this method.

In this study, there were no patients with severe cardiac and pulmonary diseases and smokers. The WR of the patients with severe cardiac diseases and pulmonary diseases cannot be obtained accurately due to circulatory disorders (6, 8). Since the data by an arterial blood gas analysis is related with the WR (21), the WR with these patients would be corrected by using the blood gas analysis data. Therefore, further investigation is required in order to determine the influence of severe cardiac and/or pulmonary diseases on this method.

We developed a fully automatic rCBF analysis program for the SIMS method (2). This program can be easily converted to the SIARG method and combined with the SIMS program; therefore, rCBF values obtained with the SIARG and SIMS methods were simultaneously checked in clinical practice in a few minutes. These fully automatic SIARG and SIMS programs improve repeatability and reproducibility of the ROIs setting for the calculation input function and rCBF values. Therefore, this program can be contributory to switch to the SIARG method. This is an advantage in applying clinical practice for many facilities using SIARG method.

## Conclusion

The results of our data suggest that converting to the SIARG method in routine clinical practice would be a rather seamless transition when used in non-smoking patients without cardiac or pulmonary diseases. However, further studies are required to determine whether this method would be applicable in other conditions not addressed by our study.
